# Comparative Evaluation of Genetically Encoded Voltage Indicators

**DOI:** 10.1016/j.celrep.2018.12.088

**Published:** 2019-01-15

**Authors:** Yuki Bando, Masayuki Sakamoto, Samuel Kim, Inbal Ayzenshtat, Rafael Yuste

**Affiliations:** 1 NeuroTechnology Center, Department of Biological Sciences, Columbia University, New York, NY 10027, USA; 2 Present address: Department of Organ and Tissue Anatomy, Hamamatsu University School of Medicine, Hamamatsu, Shizuoka 431-3192, Japan; 3 Present address: Department of Neurochemistry, Graduate School of Medicine, The University of Tokyo, Tokyo 113-0033, Japan; 4 These authors contributed equally; 5 Lead Contact

## Abstract

Imaging voltage using fluorescent-based sensors could be an ideal technique to probe neural circuits with high spatiotemporal resolution. However, due to insufficient signal-to-noise ratio (SNR), imaging membrane potential in mammalian preparations is still challenging. In recent years, many genetically encoded voltage indicators (GEVIs) have been developed. To compare them and guide decisions on which GEVI to use, we have characterized side by side the performance of eight GEVIs that represent different families of molecular constructs. We tested GEVIs *in vitro* with 1-photon imaging and *in vivo* with 1-photon wide-field imaging and 2-photon imaging. We find that QuasAr2 exhibited the best performance *in vitro*, whereas only ArcLight-MT could be used to reliably detect electrical activity *in vivo* with 2-photon excitation. No single GEVI was ideal for every experiment. These results provide a guide for choosing optimal GEVIs for specific applications.

## In Brief

Bando et al. compare the performance of recently developed genetically encoded voltage indicators (GEVIs) under multiple experimental conditions *in vitro* and *in vivo*. Each indicator has advantages and disadvantages, and no single GEVI is ideal for every experiment. The results provide a guide for choosing optimal GEVIs for specific applications.

## Graphical Abstract

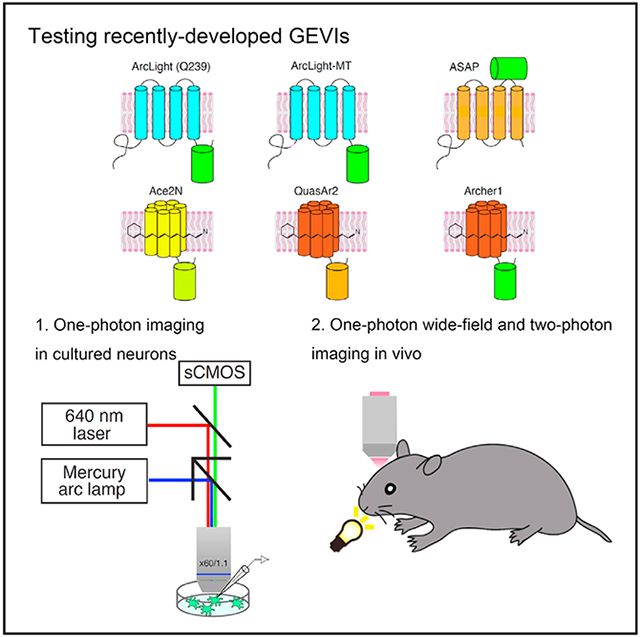

## INTRODUCTION

Probing functional neural circuits at high spatiotemporal resolution is crucial to understanding how neuronal populations work together to generate internal brain states and behavior. To do this, it is necessary to measure neural activity simultaneously in populations of neurons. Because somatic calcium influx is directly coupled with action potentials (Smetters et al., 1999), the activity of large numbers of neurons can be monitored simultaneously using calcium imaging as an indirect measure of neuronal firing with excellent signal-to-noise ratio (SNR) and without averaging (Yuste and Katz, 1991; Grienberger and Konnerth, 2012). Because of this, both calcium-sensitive organic dyes (Grynkiewicz et al., 1985; Kerr et al., 2005; Tischbirek et al., 2015) and genetically encoded calcium sensors (Chen et al., 2013; Inoue et al., 2015; Nagai et al., 2001) have been broadly used. With calcium imaging, it is possible to measure spiking activity from thousands of neurons in neural circuits with single-cell resolution (Ahrens et al., 2013; Cossart et al., 2003), and, in small transparent preparations such as *Hydra*, even capturing essentially all of the activity of the entire nervous system of a behaving animal (Dupre and Yuste, 2017).

However, calcium dynamics are not a direct proxy of membrane potential, and calcium imaging is limited in its provision of a complete description of the neuronal activity. First, somatic calcium imaging reports only action potentials (Smetters et al., 1999). Subthreshold excitatory or inhibitory synaptic inputs are essentially invisible in somatic calcium signals, making it difficult to monitor the relation between input and output of a neuron. Second, due to biophysical constrains, calcium dynamics are significantly slower than the timescale of membrane potential dynamics. Thus, when neurons fire a burst of spikes at >40 Hz, it is difficult to assess the number of spikes and spike times quantitatively with population calcium imaging (Smetters et al., 1999). Finally, calcium dynamics are shaped by complicated interactions between ionic diffusion and extrusion and intrinsic and extrinsic calcium buffers, and, moreover, calcium indicators themselves significantly alter these dynamics (Neher, 1998). For these reasons, calcium imaging is not an ideal method to measure neural activity faithfully.

Voltage imaging, however, can directly monitor membrane potential dynamics (Peterka et al., 2011; Storace et al., 2016). While voltage-sensitive dyes have been used to measure neural dynamics for >3 decades (Grinvald et al., 1981; Gross and Loew, 1989; Manita et al., 2015; Miller et al., 2012; Petersen et al., 2003; Popovic et al., 2015; Ross et al., 1977), SNR and spatial resolution have remained poor, particularly for mammalian preparations. In the last few years, intensive efforts have been made to develop genetically encoded voltage indicators (GEVIs), resulting in improved performance (Akemann et al., 2010; Flytzanis et al., 2014; Gong et al., 2015; Hochbaum et al., 2014; Jin et al., 2012; Piao et al., 2015; St-Pierre et al., 2014; Tsutsui et al., 2013). Furthermore, genetic indicators can be targeted to specific cell types or particular subcellular compartments. Newer GEVIs can detect subthreshold activity that is invisible to calcium imaging *in vivo* as well as *in vitro* (Gong et al., 2015), making it possible to generate more accurate measurements of brain functions. Therefore, as a next-generation imaging technology, voltage imaging with GEVIs appears to be a powerful tool that can supersede calcium imaging in neuroscience.

Recently developed GEVIs can be classified into 3 classes based on their molecular structure and voltage-sensing mechanism: (1) voltage-sensitive domain (VSD)-based sensors, (2) rhodopsin-based sensors, and (3) rhodopsin-fluorescence resonance energy transfer (FRET) sensors. The mechanism of sensing voltage and their characteristics (i.e., kinetics, brightness, photostability) vary among GEVIs (Inagaki and Nagai, 2016; St-Pierre et al., 2015). Therefore, it is important to understand the pros and cons of each GEVI. However, there is no systematic study comparing existing GEVIs under the same experimental conditions. In addition, most GEVI experiments have been performed by *in vitro* systems using HEK cell lines and cultured neurons. At the same time, one of the important benchmarks for these indicators is the measurement of voltage response to natural sensory stimuli *in vivo*. To perform these experiments in living brain circuits in 3 dimensions while preserving single-cell resolution, 2-photon excitation appears to be necessary.

To provide consistent data for comparison among GEVIs as a third party, we investigated side by side several recently developed GEVIs, measuring their ability to report action potentials in cultured neurons with 1-photon imaging. Then, using a fast scanning 2-photon microscope, we characterized the capacity of recent GEVIs to detect sensory stimuli in primary visual cortices *in vivo*. In our analysis, QuasAr2 demonstrated the best performance in *in vitro* experiments with 1-photon excitation. However, ArcLight-MT could detect the electrical activity of neurons in response to sensory stimulation *in vivo* with 2-photon excitation. These results provide a guide for choosing the optimal GEVIs for specific applications.

## RESULTS

### Experimental Condition *In Vitro*

**Figure 1.**
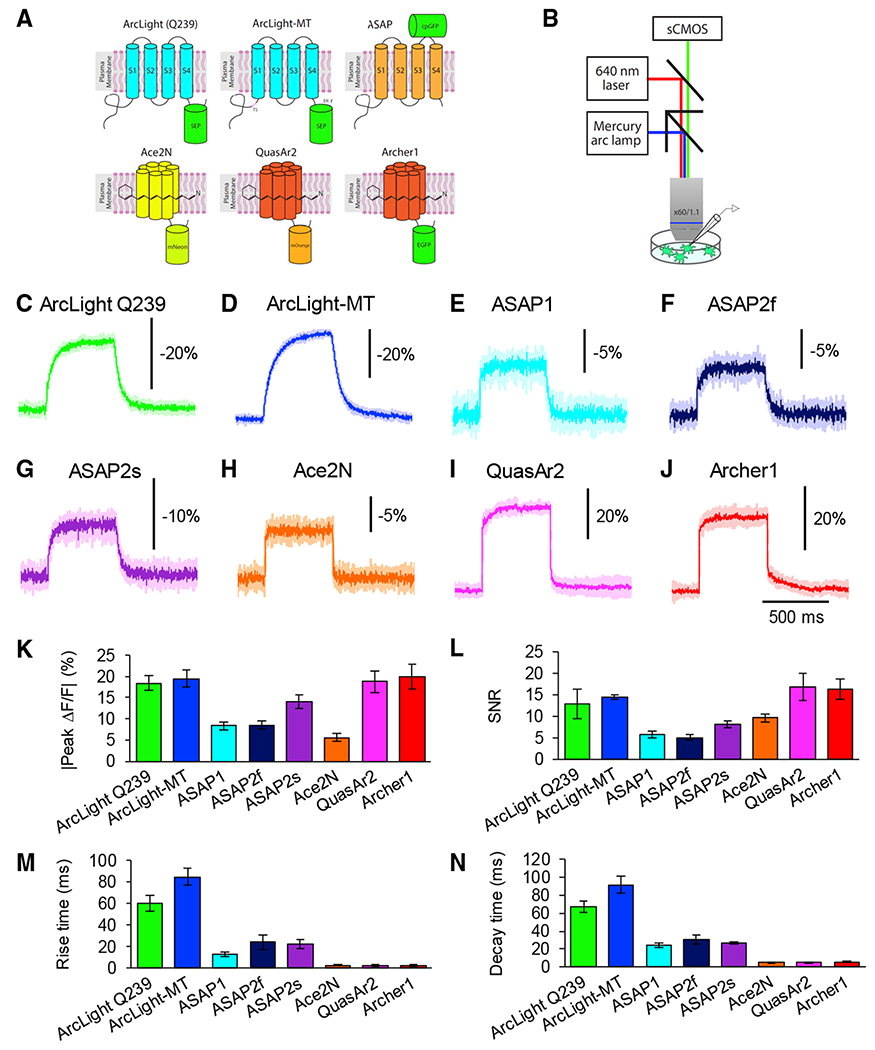
Optical Responses to Depolarizing Voltage Steps with GEVIs *In Vitro* (A) Schematic drawings of voltage indicators. ER, endoplasmic reticulum export sequence; TS, Golgi export trafficking signal. (B) Schematic drawing of voltage imaging strategy with 1-photon microscopy *in vitro*. (C–J) Representative averaged optical traces of ArcLight Q239 (C), ArcLight-MT (D), ASAP1 (E), ASAP2f (F), ASAP2s (G), Ace2N-4AA-mNeon (H), QuasAr2 (I), and Archerl (J) during depolarizing steps (from −70 to 30 mV). Five trials were averaged for each neuron. Shaded area represents SD of the mean. (K–N) Peak amplitude (K), SNR (L), rise time constants (M), and decay time constants (N) of ArcLight Q239 (n = 5 cells), ArcLight-MT (n = 5 cells), ASAP1 (n = 6 cells), ASAP2f (n = 5 cells), ASAP2s (n = 5 cells), Ace2N-4AA-mNeon (n = 5 cells), QuasAr2 (n = 4 cells), and Archerl (n = 5 cells) from the single exponential fits. Means ± SEMs are presented.

**Figure 2.**
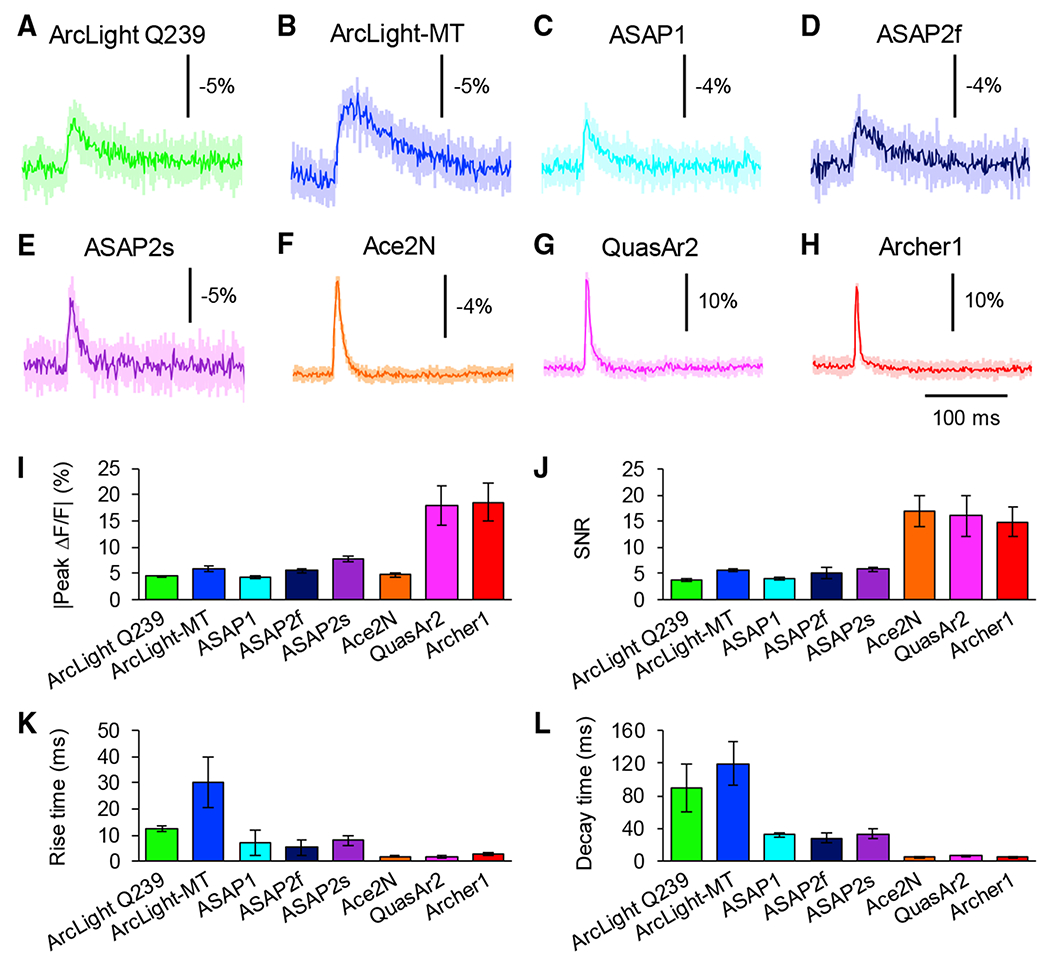
Imaging Single Action Potential Responses *In Vitro* (A–H) Average optical waveform of ArcLight Q239 (A), ArcLight-MT (B), ASAP1 (C), ASAP2f (D), ASAP2s (E), Ace2N-4AA-mNeon (F), QuasAr2 (G), and Archerl (H) in response to single action potentials induced by current injections. Ten trials were averaged for each neuron. Shaded areas represent the SD of the mean. (I–L) Comparison of GEVIs’ peak amplitude (I), SNR (J), rise time (K), and decay time (L) of ArcLight Q239 (n = 5 cells), ArcLight-MT (n = 6 cells), ASAP1 (n = 6 cells), ASAP2f (n = 5 cells), ASAP2s (n = 6 cells), Ace2N-4AA-mNeon (n = 5 cells), QuasAr2 (n = 5 cells), and Archerl (n = 5 cells). Means ± SEMs are presented. See also Figures S1, S2, and S3.

**Figure 3.**
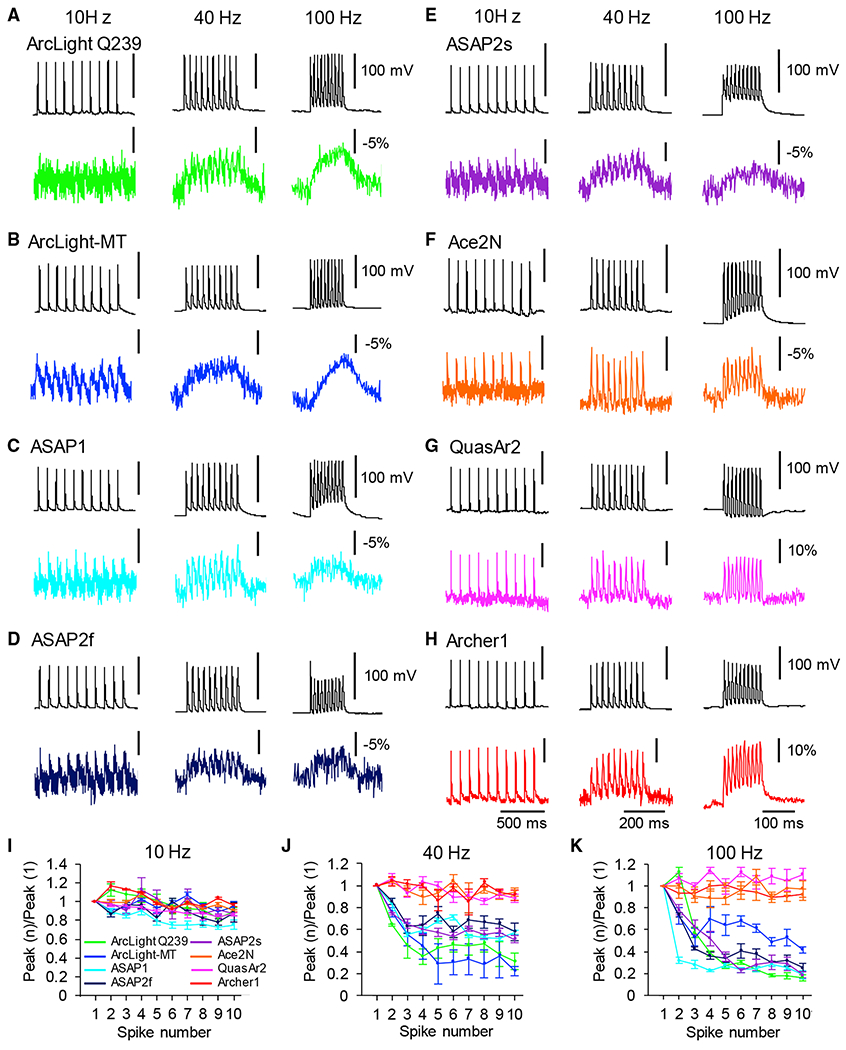
Imaging Spike Trains Responses of GEVIs *In Vitro* (A–H) Representative single electrical (top) and optical traces (bottom) in response to 10 Hz (left), 40 Hz (center), and 100 Hz (right) series of 10 action potentials. Optical traces of ArcLight Q239 (A), ArcLight-MT (B), ASAP1 (C), ASAP2f (D), ASAP2s (E), Ace2N-4AA-mNeon (F), QuasAr2 (G), and Archer1 (H) are shown. (I–K) Ratio of peak amplitude of GEVIs’ responses (means ± SEMs) as a function of spike number during trains of spikes evoked at 10 Hz (I), 40 Hz (J), and 100 Hz (K) (n = 10 trials of each GEVI).

To compare GEVI performance in an *in vitro* system, we carried out simultaneous 1-photon voltage imaging and whole-cell patch-clamp recordings from cultured hippocampal neurons expressing GEVIs (Figure 1A). For these experiments, we chose 8 GEVIs with reported good performance; 5 VSD-based sensors: ArcLight Q239 (Jin et al., 2012), a codon-optimized ArcLight Q239 with endoplasmic reticulum (ER)-export signal and Golgi-export trafficking signal (TS) sequences (hereafter called ArcLight-MT) (Kwon et al., 2017), ASAP1 (St-Pierre et al., 2014), ASAP2f (Yang et al., 2016), and ASAP2s (Chamberland et al., 2017); 2 rhodopsin-based sensors: QuasAr2 (Hochbaum et al., 2014) and Archerl (Flytzanis et al., 2014); and 1 rhodopsin-FRET sensor: Ace2N-4AA-mNeon (Gong et al., 2015). We transfected GEVIs into cultured neurons with calcium phosphate at 7 days *in vitro* (DIV 7), and performed voltage-clamp (Figure 1) and current-clamp experiments (Figures 2 and 3) between DIV 10 and 14. We imaged the fluorescence of GEVIs with an upright fluorescence microscope using a mercury arc lamp for ArcLight Q239, ArcLight-MT, ASAP1, and Ace2N-4AA-mNeon, or a 640-nm 1-photon laser for QuasAr2 and Archerl, with a 60×/1.1 N.A. (numerical aperture) objective, and a scientific complementary metal oxide semiconductor (sCMOS) camera (frame rate, 1 kHz) (Figure 1B).

### Performance of GEVIs in Response to Depolarizing Voltage Steps *In Vitro*

We characterized the response of GEVIs to depolarizing voltage steps from −70 to +30 mV for 500 ms, in voltage-clamp mode (Figures 1C–1J). In our experimental condition, ArcLight Q239, ArcLight-MT, QuasAr2, and Archerl showed similarly large responses to voltage steps of 100 mV (Figure 1K). Among these 4 indicators, QuasAr2 and Archerl showed faster rise and decay times, whereas ArcLight Q239 and ArcLight-MT had slower kinetics (Figures 1M and 1N). In contrast, ASAP1 and Ace2N-4AA-mNeon showed a smaller dynamic range. Although the signal amplitude of Ace2N-4AA-mNeon was smaller than that of QuasAr2 and Archerl, the kinetics were comparable to those of QuasAr2 and Archerl (Figures 1K, 1M, and 1N).

### Detection of Single Action Potentials with 1-Photon Microscopy *In Vitro*

We tested the performance of GEVIs for measuring single action potentials (APs) in cultured neurons. We induced APs by current injection for 5 ms in current-clamp mode and recorded optical signals (Figure 2). Two rhodopsin-based sensors, QuasAr2 and Archerl, demonstrated the best performance for the detection of single APs in terms of signal amplitude, kinetics, and SNR (Figures 2G–2L and S1). The signal amplitude of Ace2N-4AA-mNeon was smaller than that of QuasAr2 and Archerl, but the SNR and kinetics were comparable to those of QuasAr2 and Archerl (Figures 2F, 2I–L, and S1).

In contrast to rhodopsin-based and rhodopsin-FRET GEVIs, VSD-based sensors demonstrated lesser performance in the detection of single APs; peak amplitude, kinetics, and SNR were overall more limited than in rhodopsin-based and rhodopsin-FRET sensors (Figures 2 and S1). We noted that ArcLight-MT showed higher peak ΔF/F and SNR in response to single APs than ArcLight Q239 (Figures 2 and S2), when we compared the ArcLight family.

Our results showed that QuasAr2 has the best performance among 8 GEVIs *in vitro*. We then tested whether rhodopsin-based sensors can detect electrical signals in subcellular compartments from cultured neurons such as dendritic spines. We induced APs with short current pulses as described above, and recorded the optical signal of QuasAr2 from dendritic spines and their parent dendrites (Figure S3). We found that QuasAr2 could effectively detect back-propagating APs (bAPs) with high SNR and good temporal resolution. The average ΔF/F of bAPs in spines was identical to that of parent dendrites, regardless of the size of their neck lengths and head volumes (Figure S3). These results are in agreement with previous studies measuring voltage in spines with voltage-sensitive organic dyes, second harmonic generation (SHG), and nanopipette intracellular recordings (Acker et al., 2011; Holthoff et al., 2010; Jayant et al., 2017; Nuriya et al., 2006; Palmer and Stuart, 2009; Popovic et al., 2014).

### High-Frequency Spike Trains Analysis

A critical benchmark for GEVIs is to detect trains of APs and their ability to resolve individual spikes and fast spike trains in single trials, something that is difficult with calcium indicators due to the slow kinetics of the calcium response (Smetters et al., 1999). We thus tested the ability of GEVIs to track spike trains at 10, 40, and 100 Hz (Figure 3). Ace-2N-4AA-mNeon, QuasAr2, and Archer1 faithfully followed spike trains of up to 100 Hz with clear identification of individual peaks in single trials without averaging (Figures 3F–3K). In contrast, the optical traces of VSD-based GEVIs at 100 Hz had elevated baselines with poor peak detection in single trials (Figures 3A–3E and 3I–3K). At 40 Hz, ASAP1, ASAP2f, and ASAP2s followed the electrical recording more closely compared to ArcLight Q239 and ArcLight-MT.

### Photostability Analysis *In Vitro*

**Figure 4.**
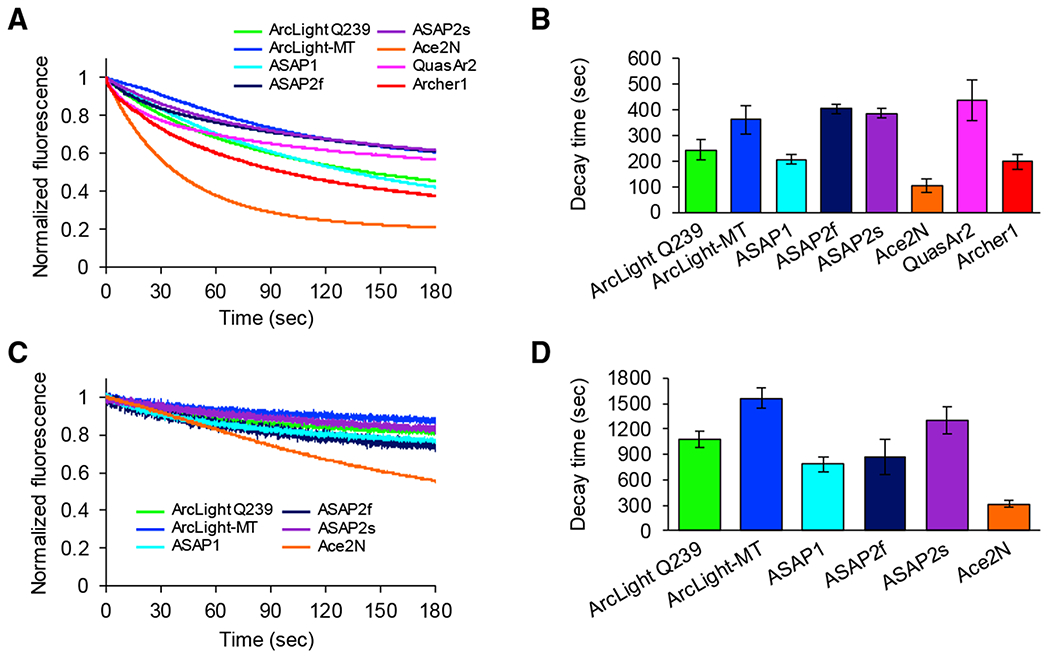
Photostability Analysis of GEVIs (A) One-photon photobleaching kinetics of GEVIs. Cultured neurons expressing ArcLight Q239 (n = 6 cells), ArcLight-MT (n = 6 cells), ASAP1 (n = 6 cells), ASAP2f (n = 7 cells), ASAP2s (n = 9 cells), Ace2N-4AA-mNeon (n = 7 cells), QuasAr2 (n = 11 cells), and Archer1 (n = 8 cells) are continuously illuminated with a mercury arc lamp. (B) Time constants by 1-photon illumination (means ± SEMs). (C) Two-photon photobleaching kinetics of GEVIs. Cultured neurons expressing ArcLight Q239 (n = 8 cells), ArcLight-MT (n = 7 cells), ASAP1 (n = 6 cells), ASAP2f (n = 9 cells), ASAP2s (n = 11 cells), and Ace2N-4AA-mNeon (n = 8 cells) are continuously scanned at 30 Hz. (D) Time constants by 2-photon illumination (means ± SEMs). See also Figure S2

One of the major considerations in voltage imaging is fluorophore photostability for long-term imaging. Therefore, we measured the photobleaching rate to characterize the photostability of each GEVI under continuous illumination, for 3 min at our imaging intensity with single-photon excitation. ArcLight-MT, ASAP2f, ASAP2s, and QuasAr2 displayed higher photostability among 8 GEVIs (Figure 4). In contrast, Ace2N-4AA-mNeon showed the fastest bleaching. In these experiments *in vitro*, we also tested the photostability of 6 GEVIs (ArcLight Q239, ArcLight-MT, ASAP1, ASAP2f, ASAP2s, and Ace2N-4AA-mNeon) with 2-photon illumination. The tendency of 2-photon photobleaching was similar to the results of 1-photon photobleaching (Figure 4).

### Voltage Imaging *In Vivo*

*In vivo* imaging is a powerful method for understanding the functional organization of neural circuits (Grienberger and Konnerth, 2012; Grinvald et al., 1988). Thus, *in vivo* measurement of natural sensory-evoked responses using GEVIs appears to be a critical benchmark. Although some of the GEVIs have been used in living animals (Akemann et al., 2013; Storace et al., 2015), comparative evaluations of different GEVIs under the same experimental conditions with cellular resolution *in vivo* have not been performed to our knowledge. To do so, we set out to determine whether GEVIs could detect sensory-evoked electrical activity *in vivo* with 1- or 2-photon microscopy, under identical experimental conditions (Figure S4).

To express GEVIs in the mouse visual cortex efficiently, we electroporated a plasmid encoding GEVIs under the control of CAG promoters in mouse embryos at embryonic day 15.5 (E15.5) using a triple electrode (dal Maschio et al., 2012) (Figure S4). Under those conditions, all GEVIs localized efficiently to the plasma membrane of the somata and dendrites of pyramidal neurons *in vivo* (Figure 6A). We then examined whether GEVIs could be used to detect visually evoked electrical activity in layer 2/3 of the primary visual cortex (V1) in anesthetized, head-fixed mice between postnatal days 35 and 56 (P35-P56) *in vivo* with 1- or 2-photon microscopy, using a 10-ms flash of light as visual stimulation. We collected high-speed fluorescent movies (frame rate, 30 Hz, 128 × 128 pixels for 1-photon wide-field imaging, and frame rate, 116 Hz, 128 × 128 pixels for 2-photon imaging) over a field of view using a 4×/0.28 N.A. objective lens and an sCMOS camera (1-photon wide-field imaging) or a 25×/1.05 N.A. objective and a resonant scanner (2-photon imaging). We tested 4 GEVIs–ArcLight-MT, ASAP1, ASAP2s, and Ace2N-4AA-mNeon–and compared their performance with EGFP, a voltage-insensitive fluorescent protein. We excluded ASAP2f, because its performance with 1-photon imaging *in vitro* was similar to that of ASAP1. We also excluded rhodopsin-based GEVIs (QuasAr2 and Archer1) because these sensors were unfortunately too dim under our 1-photon excitation with light-emitting diodes (LEDs) or 2-photon excitation conditions (Brinks et al., 2015). We considered that visually evoked potentials were detectable with GEVIs if the SNRs of GEVIs were significantly larger than those of control EGFP-labeled cells (see below).

### One-Photon Wide-Field Imaging *In Vivo*

**Figure 5.**
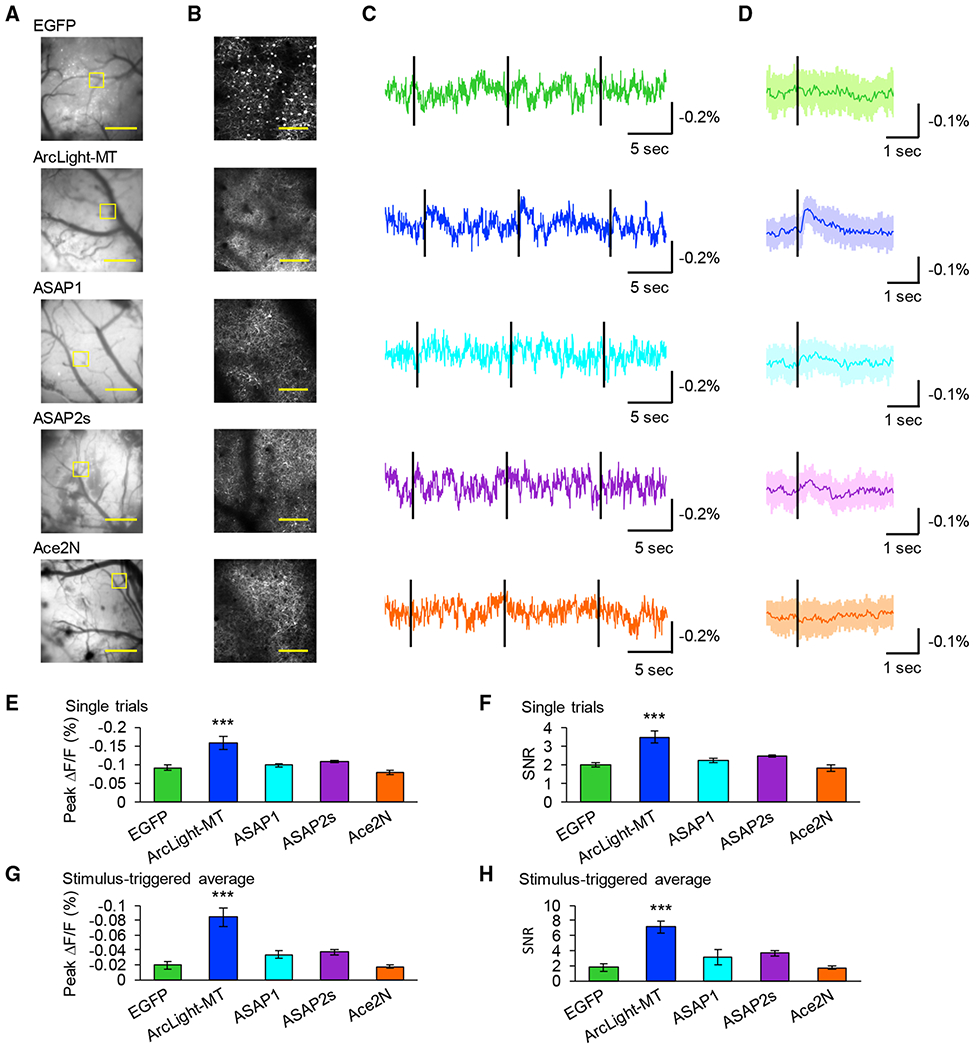
One-Photon Wide-Field Voltage Imaging *In Vivo* (A) One-photon images of the primary visual cortex expressing GEVIs or EGFP. Scale bar, 1 mm. (B) Two-photon images of the areas shown with the yellow square in (A). Scale bar, 100 μm. (C) Representative single-trial optical traces. Black lines indicate timing of visual stimulus using flash of light illumination for 10 ms. (D) Average visually evoked optical response over 10 trials. Black lines indicate the timing of the visual stimulus. (E and F) Peak fluorescence change (E) and SNR (F) of single trials measured with each GEVI or EGFP. Means ± SEMs are presented. (G and H) Peak fluorescence change (G) and SNR(H) of stimulus-triggered average. Means ± SEMs are presented, n = 5 field of views (FOVs) (EGFP, Ace2N-4AA-mNeon), 6 FOVs (ArcLight-MT, ASAP1, ASAP2s). One FOV was imaged in 1 mouse. ***p < 0.001 compared with EGFP, Dunnett test. See also Figure S4.

Voltage imaging *in vivo* has been mostly performed using 1-photon wide-field imaging (Akemann et al., 2010; Borden et al., 2017; Carandini et al., 2015; Liang et al., 2015; Marshall et al., 2016; Tsutsui et al., 2013). In some of these experimental conditions, optical signals were correlated with the local field potential (LFP) (Borden et al., 2017; Carandini et al., 2015; Liang et al., 2015; Marshall et al., 2016). We tested recently developed GEVIs with 1-photon wide-field imaging from the primary visual cortex *in vivo*. Before or after wide-field imaging, we also performed 2-photon imaging and confirmed that GEVIs or EGFP were expressed in the field of views (FOVs) (Figures 5A and 5B). Recently, it was reported that imaging scattering of green light can detect spontaneous or sensory-evoked hemodynamic signals (Bouchard et al., 2009; Ma et al., 2016). Therefore, we first tested whether a hemodynamic signal could be detected with our system by imaging EGFP, a voltage-insensitive GFP. As a result, visually evoked fluorescence change was not detected with EGFP, suggesting that the hemodynamic signal was not significant with our wide-field imaging system (Figures 5C and 5D). Next, we imaged ArcLight-MT, ASAP1, ASAP2s, Ace2N, and calculated peak ΔF/F and SNR of both single trials and average traces. We found that ArcLight-MT detected visually evoked signals in both single trials and stimulus-triggered averages (Figures 5C and 5D). In single trials (Figures 5E and 5F), ArcLight-MT showed significantly larger peak ΔF/F and SNR than EGFP, and in stimulus-triggered averages (Figures 5G and 5H), it showed significantly larger peak amplitudes and SNRs. ASAP1, ASAP2s, and Ace2N did not show significant optical signals in response to visual stimuli in either single trials or averages (Figures 5C–5H). These results indicate that only ArcLight-MT can reliably detect visually evoked potentials with 1-photon wide-field imaging *in vivo*.

### Measuring Electrical Activity with Cellular Resolution *In Vivo*

**Figure 6.**
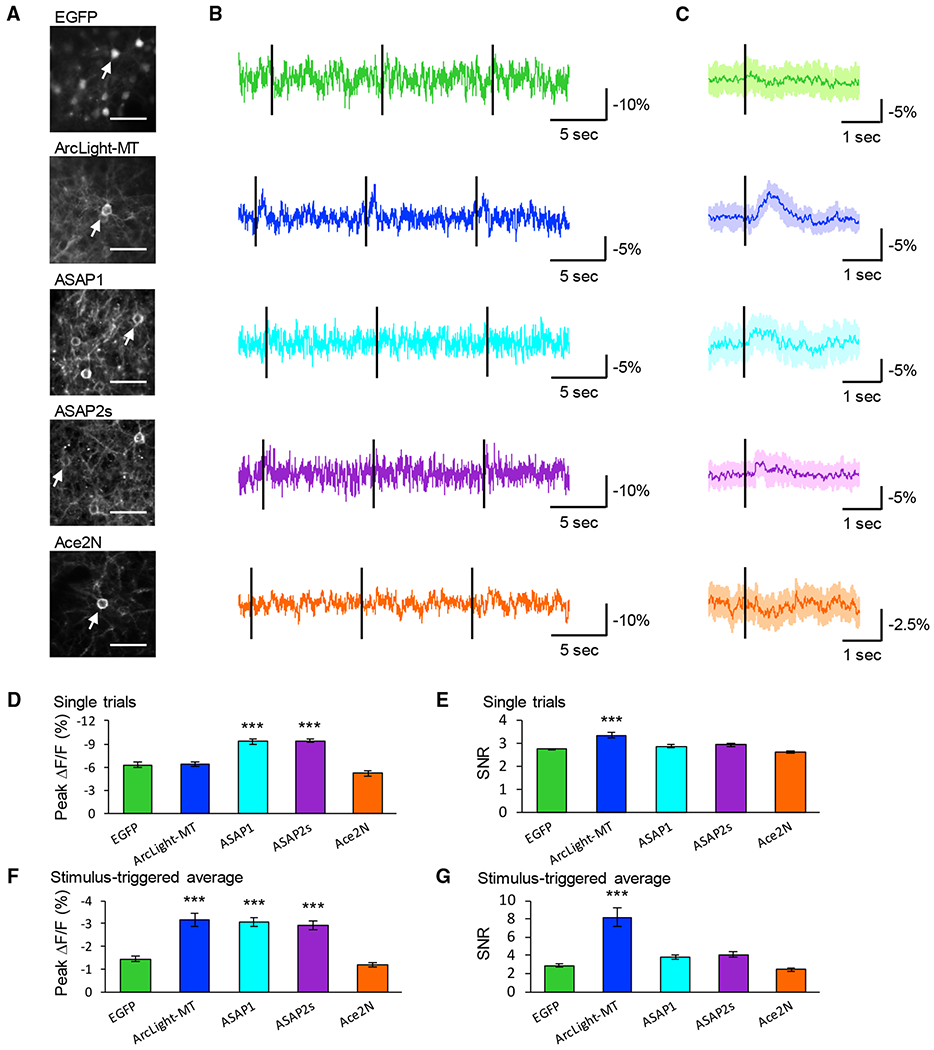
Two-Photon Voltage Imaging with Cellular Resolution *In Vivo* (A) Two-photon images of layer 2/3 pyramidal neurons in the visual cortex expressing GEVIs or EGFP *in vivo*. Scale bar, 40 μm. Arrows indicate cells whose optical signals are shown in (B) and (C). (B) Representative traces of each GEVI. Black lines indicate the timing of the visual stimuli. Ten visual stimuli were applied during each recording. (C) Average GEVI optical waveform in response to visual stimulus over 10 trials, with the shaded area representing the SD of the mean. Black lines indicate the timing of the visual stimuli. (D and E) Peak amplitude (D) and SNR (E) of single trials. Means ± SEMs are presented. (F and G) Peak amplitude (F) and SNR (G) of stimulus-triggered average. Means ± SEMs are presented, n = 18 cells from 5 mice (EGFP), 12 cells from 5 mice (ArcLight-MT), 19 cells from 7 mice (ASAP1), 20 cells from 6 mice (ASAP2s), and 25 cells from 5 mice (Ace2N-4AA-mNeon). ***p < 0.001 compared with EGFP. Dunnett test. See also Figures S4, S5, and S6.

To test the performance of GEVIs *in vivo*, in control experiments we recorded and characterized visually evoked activity from layer 2/3 pyramidal neurons using whole-cell patch-clamp recordings *in vivo*. Consistent with previous reports, all of the trials showed subthreshold depolarization and some trials included APs in response to visual stimuli with a flash of light of 10 ms (Funayama et al., 2015) Figure S5). Because of the variation of spike timing, APs were removed and subthreshold synaptic potentials were extracted after averaging over 10 trials (Figures S5C and S5D). We then performed 2-photon voltage imaging and recorded visually evoked optical responses (Figure 6). In single trials (Figures 6D and 6E), ArcLight-MT showed a significantly larger SNR than EGFP, suggesting that the visually evoked potential is detectable from single trials at cellular resolution with ArcLight-MT. In similar single trial experiments, the peak ΔF/F of ASAP1 and ASAP2s were also significantly larger than EGFP, but the SNR of ASAP1 and ASAP2s were not significantly larger than EGFP. Thus, the large ΔF/F of ASAP1 and ASAP2s was presumably due to large baseline noise. In stimulus-triggered averages (Figures 6F and 6G), ArcLight-MT, ASAP1, and ASAP2s showed significantly larger peak ΔF/F, and ArcLight-MT showed a significantly larger SNR than EGFP. These results indicate that only ArcLight-MT can detect visually evoked potential, presumably subthreshold depolarization with cellular resolution.

Because ASAP1, ASAP2s, and Ace2N-4AA-mNeon showed high SNRs and temporal resolutions for single AP detection by single photon excitation *in vitro*, we also examined whether they could detect spikes with 2-photon imaging *in vivo*. It was unfortunate that none of these indicators showed optical signals in response to APs, even after averaging (Figure S6).

### Detection of Optical Field Potentials with GEVIs *In Vivo*

**Figure 7.**
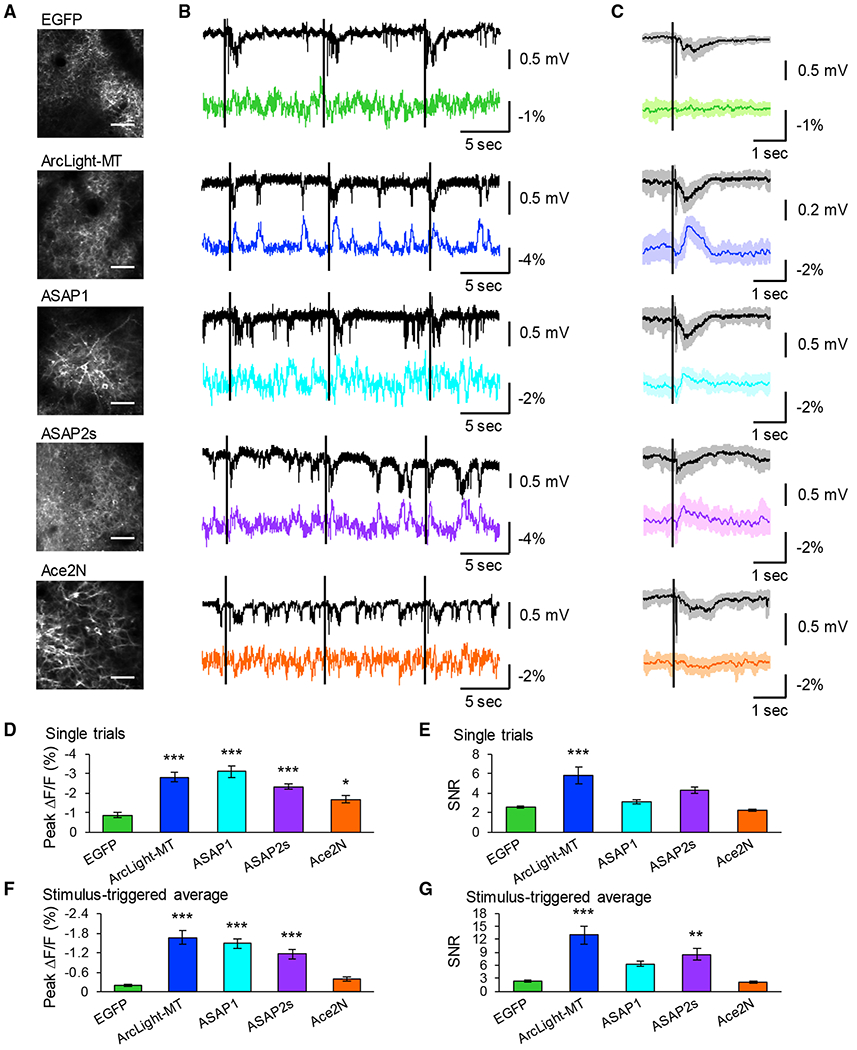
Two-Photon Optical Field Potential Imaging with GEVIs (A) Two-photon images of primary visual cortex expressing GEVIs. Scale bar, 50 μm. (B) Representative traces of LFPs (black traces) and OFPs (colored traces). Fluorescent changes were imaged from all FOVs as a population. Black lines indicate the timing of the visual stimuli. (C) Average LFP (black traces) and optical waveform (colored traces) in response to visual stimuli over 10 trials, with shaded areas representing SD of the mean. The black lines indicate the timing of the visual stimuli. (D and E) Peak amplitude (D) and SNR (E) of single trials. Means ± SEMs are presented. (F and G) Peak amplitude (F) and SNR (G) of stimulus-triggered average. Means ± SEMs are presented, n = 10 FOVs from 5 mice (EGFP), 11 FOVs from 6 mice (ArcLight-MT), 9 FOVs from 7 mice (ASAP1), 9 FOVs from 4 mice (ASAP2s), and 12 FOVs from 6 mice (Ace2N-4AA-mNeon). **p < 0.01, ***p < 0.001 compared with EGFP. Dunnett test. See also Figure S7.

To examine whether GEVIs can also be used to detect LFP-related signals with 2-photon imaging *in vivo*, we recorded visually evoked LFPs and performed 2-photon voltage imaging simultaneously *in vivo*. In all of the recordings, visually evoked potentials were detected with LFPs (Figures 7B and 7C). For analysis, we took the entire FOV as a region of interest (ROI), so that optical signals reported the average synaptic inputs from somata, dendrites, and axons, which could presumably reflect LFPs (we refer to this analysis as optical field potential [OFP]). We confirmed that EGFP did not show visually evoked fluorescence change, suggesting that the hemodynamic signal was not significant in 2-photon imaging (Figures 7A–7C). We found that only ArcLight-MT could detect LFP as an OFP signal without averaging (Figures 7B–7E). After averaging over 10 trials, ArcLight-MT and ASAP2s could detect visually evoked potentials (Figures 7C, 7F, and 7G). Also, the optical signal of ArcLight-MT was correlated with spontaneous LFP (Figure 7B). ASAP1 showed a slightly larger SNR of stimulus-triggered averages, and ASAP2s showed slightly larger SNRs of single trials than EGFP, although they are not statistically significant.

### Two-Photon Voltage Imaging with Slower Scanning Rate *In Vivo*

Our *in vitro* study with 1-photon microscopy demonstrated that ArcLight constructs have slower kinetics than other types of indicators. In addition, sensory-evoked subthreshold events are slower than APs. Therefore, signals of ArcLight-MT could be detected with slow sampling rates. To explore this, we performed *in vivo* 2-photon voltage imaging at a slower frame rate (30 Hz, 512 × 512 pixels). We focused on ArcLight-MT for this experiment because it showed the best performance with *in vivo* 2-photon imaging among 4 indicators. We recorded OFPs of ArcLight-MT with 2-photon imaging and analyzed stimulus-triggered averaged responses. ArcLight-MT could detect sensory-evoked OFP with a 30-Hz scanning rate without averaging, which is similar to fast scanning experiments (Figure S7). This result suggests that it is important to not only choose an appropriate GEVI but also match it with an appropriate scanning rate, depending on the properties of the GEVI, the type of electrical events, and regions of the neuron to record.

## DISCUSSION

We compared 8 different recently developed GEVIs under similar optical and experimental paradigms. Under experimental conditions that are widely used for functional imaging *in vitro* using 1-photon excitation, we could detect clear optical signals in response to depolarizing voltage steps and APs with all of the tested GEVIs. Although ArcLight Q239 and ArcLight-MT demonstrated a high dynamic range in voltage steps, their peak amplitudes in response to APs were limited due to their slow kinetics. Conversely, using a rhodopsin-FRET sensor, Ace2N-4AA-mNeon, we could detect single APs and spike trains with high temporal resolution with high SNR using standard mercury arc lamp illumination. However, Ace2N-4AA-mNeon also showed the fastest photobleaching among the 8 indicators. Thus, further improvement in the photostability of rhodopsin-FRET sensors appears to be necessary for long-term imaging. A rhodopsin-based GEVI, QuasAr2, showed the highest signal amplitude, SNR, and temporal resolution *in vitro*. Furthermore, QuasAr2 could detect electrical activity from dendritic spines that cannot be easily accessed by the conventional methods of electrophysiology. QuasAr2 thus seems the ideal choice for the optical detection of single APs from bursts of spikes using 1-photon imaging *in vitro*.

We also compared the performance of 4 GEVIs *in vivo*. Using fast-scanning 2-photon microscopy, we demonstrated the feasibility of imaging membrane potential dynamics with single-cell resolution in the mammalian cortex. Our results showed that ArcLight-MT was the most sensitive GEVI to measure the responses to visual or somatosensory stimulation, although it was still difficult overall to extract reliable signals from individual trials. This is consistent with a previous study, which reported that ArcLight A242 can record the population signals from dendritic tufts of mitral cells and tufted cells in the mouse olfactory bulb in response to odor stimulation with 2-photon imaging (Storace et al., 2015). Because ArcLight-MT has a large dynamic range, it could also be used to detect subthreshold membrane potential changes *in vivo*. The ArcLight-MT signals we measure likely reflect UP states that are invisible to calcium imaging, but further research is necessary to develop new GEVIs that can clearly discriminate both APs and subthreshold events *in vivo*. In imaging ASAP1, ASAP2s, and Ace2N-4AA-mNeon *in vivo*, we did not detect reliable optical responses to visual stimulation, in spite of its high performance with 1-photon excitation *in vitro*. This is presumably because these 3 GEVIs have less voltage sensitivity than ArcLight-MT, as shown in Figure 1, and also because their faster responses could make them harder to image, as they provide fewer integrated photons per response. Ace2N did not report any optical signals among all of the experimental conditions *in vivo*. This may be because Ace2N is made from a bacterial rhodopsin and is a FRET sensor. These properties of Ace2N may make *in vivo* imaging, especially 2-photon imaging, difficult. Further engineering of rhodopsin-based or rhodopsin-FRET sensors to optimize their 2-photon responses may be required for the precise recording of APs *in vivo*. In addition, fast scanning (>1 kHz) or scanless (Nikolenko et al., 2008) 2-photon microscopy seems necessary.

Several factors could confound the optical monitoring of membrane potential dynamics with GEVIs *in vivo*, such as fluctuation in blood oxygen level, breathing, and energy metabolism. Previously, flavin autofluorescence and hemodynamic signals were used to monitor brain activity *in vivo* (Shibuki et al., 2003; Bouchard et al., 2009; Ma et al., 2016). The autofluorescence of flavoproteins is small, and it increases when neurons become active (Shibuki et al., 2003). However, the fluorescence of the GEVIs we tested *in vivo* decreased when the neurons depolarized. Thus, flavin autofluorescence likely did not contaminate in our recordings. Hemodynamic signals can also be detected with the reflection of green light (Bouchard et al., 2009; Ma et al., 2016), so there is a possibility that the hemodynamic signal could have contaminated the signals of green GEVIs. However, we imaged EGFP, a voltage-insensitive fluorescent protein, and did not And any significant fluorescence change in response to visual stimuli in either 1-photon or 2-photon imaging *in vivo*. Therefore, we excluded the possibility that hemodynamic signals were contaminating our voltage imaging traces.

In summary, we And that there is no single GEVI that is ideal for every experiment. While QuasAr2 outperformed the other GEVIs *in vitro*, ArcLight-MT performed better under 2-photon imaging *in vivo*. Given this situation, it is critical to understand the advantages and disadvantages of each GEVI. When selecting voltage indicators for a particular application, it is important to consider several factors, including the amplitude, kinetics, and spectrum of the indicator and also the purpose and optical approach of the experiment (i.e., 1 photon or 2-photon imaging, *in vitro* or *in vivo*, detection of APs or subthreshold events). Another finding is the necessity of using 2-photon excitation while screening GEVIs for their use *in vivo*, since a good *in vitro* performance with 1-photon excitation does not necessarily guarantee an adequate response *in vivo* under 2-photon excitation.

Our comparative dataset provides a guide to which GEVI is suitable for a particular study. At the same time, the 8 GEVIs tested were chosen to represent the most promising type of constructs at the beginning of the study, but we caution the reader to interpret our study solely as a temporal snapshot of this rapidly evolving field, since newer constructs are constantly developed, and it is possible that the tested GEVI will be superseded by improved ones.

## STAR★METHODS

### CONTACT FOR REAGENT AND RESOURCES SHARING

Further information and request for reagents and resources should be addressed to, and will be fulfilled by, the Lead Contact, Yuki Bando (bando@hama-med.ac.jp).

### EXPERIMENTAL MODEL AND SUBJECT DETAILS

All procedures involving animals were in accordance with the US National Institutes of Health Guide for the care and use of laboratory animals and approved by the Institutional Animal Care and Use Committees (IACUC) of Columbia University, and Animal Care and Use Review Office (ACURO) from Army Research Office (ARO). For experiments, both sex of wild-type C57BL/6J mice (P0 for primary hippocampal cultures) or CD-1; ICR mice (E15 for *in utero* electroporation, P35 ~P60 for imaging and electrophysiology) were used. Animals were housed and maintained in a temperature-controlled environment on a 12-h light-dark cycle, with *ad libitum* food and water.

### METHOD DETAILS

#### Voltage indicators

The following GEVI were kindly shared by developers: ASAP1, ASAP2f and ASAP2s (Dr. Michael Lin), ArcLight Q239 and ArcLight-MT (Dr. Vincent Pieribone), QuasAr2 (Dr. Adam Cohen), Archer1 (Dr. Viviana Gradinaru). Ace2N-4AA-mNeon was synthesized with the same codon usage as previously described (Gong et al., 2015).

#### Voltage imaging in dissociated hippocampal neurons

Primary cultured hippocampal neurons were prepared from P0 C57BL/6J mouse pups. Hippocampal CA1 and CA3 were isolated, digested with papain (Worthington Biochemical), and plated onto 12 mm coverslips coated with poly-L-Lysine (BD Biosciences) at a density of 100,000 cells per coverslip. Cultures were maintained in Neurobasal medium (Life Technologies) containing 0.5 mM glutamine (Sigma-Aldrich), and 2% B-27 supplement (Life Technologies) and kept in an incubator at 37°C with 5% CO_2_. Cells were transfected using calcium phosphate on day *in vitro* 7 (DIV 7) with a plasmid encoding GEVIs under the control of the CMV promoter. Endotoxin-free DNA of 2 μg and 1.875 μL CaCl_2_ of 2M (final Ca^2+^ concentration 250 mM) were added in 15 μL double distilled water. Then, 15 μL of 2 × HEPES-buffered saline (pH 7.05) was added to the DNA-CaCl_2_ mixture. After 20 min incubation at room temperature, the growth medium was removed from the well and replaced with pre-warmed minimal essential medium (MEM). Then, the DNA-CaPO_4_ mixture was added to each well and incubated for 45 min at 37°C. After the transfection, each well was washed three times with 1 mL pre-warmed MEM before the original growth medium was returned.

Simultaneous voltage imaging and whole-cell patch-clamp recordings in cultured hippocampal neurons were performed between DIV 10 and 14. Coverslips with transfected cells were placed in a recording chamber that was perfused with artificial cerebrospinal fluid (ACSF) containing the following (in mM): 126 NaCl, 3 KCl, 2 CaCl_2_, 2 MgSO_4_, 1.1 NaH_2_PO_4_, 26 NaHCO_3_, saturated with 95% O_2_, 5% CO_2_. Voltage imaging was performed using Olympus BX51WI upright microscope with a 60×/1.1 N.A. water immersion objective lens (Olympus). To acquire ArcLight Q239, ArcLight-MT, ASAP1, ASAP2f and ASAP2s images with mercury arc lamp (Osram), we used a 480/40 excitation Alter (Chroma), a dichroic mirror 495lp (Chroma), and a 535/50 m emission Alter (Chroma). To acquire Ace2N-4AA-mNeon images, we used a 500/24 excitation Alter (Chroma), a dichroic mirror 520lp (Chroma), and a 542/27 m emission Alter (Chroma). To acquire Archer1 and QuasAr2 images with a one-photon laser (400 W/cm^2^, Coherent Obis 637-140LX), we used a 620/60 excitation Alter (Chroma), a dichroic mirror 650lp (Chroma), and a 705/100nm emission Alter (Chroma). Fluorescent images were captured by a sCMOS camera (Orca-Flash 4.0, Hamamatsu) in Figures 1, 2, and 3 or EM-CCD camera (ImagEM C9100-13, Hamamatsu) in Figure S3 with HC Image software (Hamamatsu). Images were acquired at 1 kHz with 1 × 1 binning (sCMOS) or 250 Hz with 2 × 2 binning (EM-CCD). Whole-cell patch-clamp recording was performed using pulled borosilicate glass pipettes 5-7 MΩ. Membrane potential was recorded at 10 kHz and Altered with a Bessel Alter above 4 kHz using an Multiclamp 700B ampliAer (Molecular Devices) and custom-made software written with LabView (http://apacker83.github.io/PackIO, National Instruments) (Packer et al., 2012). The internal solution contained the following (in mM); 120 K-gluconate, 3 KCl, 4 Mg-ATP, 0.3 Na-GTP, 20 HEPES, 14 Tris-phosphocreatine, pH 7.3. Single APs were evoked by 5 ms current injections. For spike trains experiments, 10 Hz, 40 Hz and 100Hz series of 10 APs were evoked by 2 ms current injections. For the voltage-clamp experiments, holding potential was set at −70 mV, and 5 depolarizing voltage steps to +30 mV were applied. We discarded from future analysis neurons with resting membrane potential greater than −50 mV or membrane resistance lower than 40 MΩ. We collected data from 2-3 different culture preparations (2-3 neurons were tested per a batch), and confirmed that imaging results of 8 GEVIs we tested were reproducible among different culture preparations.

#### Analysis of photostability

Cultured neurons were transfected GEVIs as described above. For one-photon excitation, neurons expressing GEVIs were continuously illuminated with a mercury arc lamp (ArcLight Q239, ArcLight-MT, ASAP1, ASAP2f, ASAP2s, Ace2N-4AA-mNeon) or a 640 nm-laser (QuasAr2, Archer1) with same procedures as described above. Images were taken at 10 Hz with a sCMOS camera (Orca-Flash 4.0, Hamamatsu) for 3 min without patching in the external solution (in mM: 145 NaCl, 5 KOH, 10 HEPES, 2 CaCl_2_, 1 MgCl_2_, 10 glucose, pH 7.3). For two-photon excitation, cultured neurons expressing GEVIs were continuously scanned at 30 Hz with Ti:sapphire laser tuned to 920 nm (ASAP1, ASAP2f, ASAP2s), 940 nm (ArcLight Q239, ArcLight-MT), 990 nm (Ace2N-4AA-mNeon). Laser power was set to 28 mW at the stage. Images were taken at 30 Hz for 3 min with a resonant scanning mode.

#### *In utero* electroporation

*In utero* electroporation experiments were conducted as previously described with some modifications (Bando et al., 2016; dal Maschio et al., 2012). Mouse embryos at embryonic day 15.5 (E15.5) were electroporated in utero with pCAG-EGFP, pCAG-Ace2N-4AA-mNeon, pCAG-ASAP1, pCAG-ASAP2s, pCAG-ArcLight-MT plasmids. Pregnant mice (CD-1; ICR, Charles River) were anesthetized with isoflurane (3% v/v for induction, 2% v/v for surgery) and the uterine horns were exposed by laparotomy. The plasmid (1.0 μg/μl in final concentration) mixed with Fast Green (0.05% w/v; Sigma-Aldrich) was injected into the left lateral ventricle of each embryo through the uterine wall using a glass micropipette pulled with a vertical puller (PP-83, Narishige). After soaking the uterine horn with warm phosphate-buffered saline (PBS, 37°C), each embryo’s head was carefully held between tweezers with platinum disk electrodes (CUY650P5, Nepa Gene) that were connected to − end, while the third electrode (CUY7004L, Nepa Gene) was accurately positioned at the visual or barrel cortex that was connected to + end. Subsequently, electric pulses were delivered through these triple-electrodes using an electroporator (NEPA21, Nepa Gene) (Figure S4). After the electroporation, the uterine horns were returned into the abdominal cavity and the skin was closed with sutures. *In vivo* imaging was conducted 5-8 weeks after birth.

#### Animal surgery for *in vivo* imaging.

Mice were anesthetized with isoflurane (3% v/v for induction, 1.5-2% v/v for surgery). A custom-made stainless steel head plate was fixed to the skull using cyanoacrylate adhesive and dental cement above the left visual cortex (centered 2.5 mm lateral to the midline and 0.3 mm anterior of lambda) and barrel cortex (centered 2.5 mm lateral to the midline and 1.5 mm posterior of bregma). Craniotomies (1.5 mm × 1.5 mm for whole-cell recordings, 2 mm × 2 mm for two-photon single-cell and OFP imaging, 3 mm × 3 mm for one-photon wide-field imaging) were made and filled with 2.0% (w/v) agarose in PBS. To suppress the motion of the exposed brain, a cover glass was placed over the agarose for imaging without electrophysiological recordings. The animal was then transferred, while maintaining anesthesia, to the animal stage under the one or two-photon microscope.

#### *In vivo* one-photon wide-field imaging

One-photon wide-field imaging was performed with BX61WI microscope (Olympus) equipped with 4 × /0.28 N.A. dry objective lens (Olympus) and sCMOS camera (Orca-Flash 4.0, Hamamatsu). For excitation of GEVIs, 470 nm fiber-coupled LED (M470F3, Thorlabs) and a dichroic mirror 495lp (Chroma) was used for EGFP, ArcLight-MT, ASAP1 and ASAP2s, and 503nm fiber-coupled LED (M505F3, Thorlabs) and a dichroic mirror 520lp (Chroma) was used for Ace2N-4AA-mNeon. Fluorescence was isolated with 535/50 m emission filter (EGFP, ArcLight-MT and ASAP2s) or 542/27 m emission filter (Ace2N-4AA-mNeon) (Chroma). During imaging, mice were head-fixed, and were anesthetized with isoflurane (1.5%–2.0% v/v). Images were acquired as images of 128 × 128 pixels (8 × 8 binning) at 30 Hz. Visual stimuli were applied to contralateral eye using a white LED flash light (10 ms) with the use of a Master-8 pulse stimulator (A.M.P.I.).

#### *In vivo* two-photon voltage imaging

*In vivo* two-photon imaging was conducted with a two-photon microscope (Prairie, Bruker), a 25×/1.05 N.A. water immersion objective (Olympus) and tunable Ti-sapphire laser (Coherent Chameleon Ultra II, 140-fs pulses, 80 MHz repetition rate). All *in vivo* voltage imaging of GEVIs-expressing neurons was performed in the layer 2/3 of the left visual or barrel cortex (approximately 150-300 μm from the pia). During imaging, mice were head-fixed, and were anesthetized with isoflurane (1.5%–2.0% v/v).

Visual stimuli were applied as described above. Whisker stimuli were applied to contralateral whiskers using a brief air puff (50 ms) with the use of a Picosprizer III (Parker). Ti:sapphire laser laser was tuned at 920 nm for ASAP1 and ASAP2s, 940 nm for EGFP and ArcLight-MT, and 990 nm for Ace2N-4AA-mNeon. Spontaneous and sensory-evoked activities were recorded at the resolution of 64 × 64 pixels with 8× zoom at 226 Hz (Figure S6), 128 × 128 pixels with 4× zoom at 116 Hz (Figure 6 ), 128 × 128 pixels with 2× zoom at 116 Hz (Figure 7) or 512 × 512 pixels without zooming at 30 Hz (Figure 5B) and 2× zoom at 30 Hz (Figure S7) with a resonant scanning mode. Fluorescent signals of EGFP, Ace2N-4AA-mNeon, ArcLight-MT, ASAP1 and ASAP2s were isolated with HQ 525/70 m-2p filter (Chroma Technology).

#### *In vivo* electrophysiology

*In vivo* two-photon-targeted electrophysiology was performed as previously reported (Carrillo-Reid et al., 2016; Margrie et al., 2003; Yang et al., 2018). During the experiment, mice were anaesthetized with isoflurane (1.5% v/v). A square cranial window of 1.5 mm was made, and the dura was carefully removed at the access point of the recording pipette. To reduce movement artifact, the cranial window was covered with 2% agarose gel in HEPES-buffered ACSF (in mM: 150 NaCl, 2.5 KCl, 10 HEPES, 2 CaCl_2_, 1 MgCl_2_, pH 7.3). For all experiments, patch pipettes of 5 ~7 MU were pulled with a horizontal puller (DMZ-Universal puller, Zeitz Instruments). All experiments were performed in the primary visual cortex (2.5 mm lateral from the lambda), and data was obtained at 10 kHz using Multiclamp 700B amplifier (Molecular Devices).

For whole-cell recording, patch pipettes were filled with internal solution (in mM: 120 K-gluconate, 3 KCl, 7 NaCl, 4 Mg-ATP, 0.3 Na-GTP, 20 HEPES, and 14 Tris-phosphocreatine, 0.025 Alexa-594, pH 7.3). High positive pressure (200-250 mBar) was applied to the patch pipettes until they were inserted into the brain, and then pressure was reduced to 30 ~40 mBar to approach a cell. Access resistance was 13 ~75 MΩ. Flash light-induced visual response was recorded with a current-clamp mode. For loose-seal cell-attached recording, glass pipettes were filled with HEPES-buffered ACSF. Spontaneous APs were recorded with a voltage-clamp mode (holding potential = 0 mV). The data was low-pass-filtered at 4 kHz. For recording of local field potential, glass pipettes were filled with HEPES-buffered ACSF. Flash light-induced visual response was recorded with current-clamp mode, and low-pass filtered at 1 kHz.

### QUANTIFICATION AND STATISTICAL ANALYSIS

Analyses were performed with MATLAB (Math Works) and ImageJ (NIH). For the *in vitro* imaging, background was subtracted with ImageJ, and bleach correction was performed with spline function in MATLAB before calculating ΔF/F. ROIs were manually selected around somata or dendritc spines in the time series averaged image. The rise time was defined as the time to peak from the beginning of the stimulus to the time point of the peak fluorescence amplitude. The decay time was defined as the time to the baseline from the time point of the peak fluorescence amplitude. SNR was quantified as peak ΔF/F over the standard deviation of the baseline fluctuation during a 50 ms period before stimulus onset. In Figure 1 the rise and decay time constants were determined by single exponential fit. In Figure 3, the rise and decay time were determined by measuring time from baseline to peak and from peak to baseline, respectively. In Figure 4, fluorescence was normalized at t = 0 and averaged over all cells. Decay curves were fit to a single exponential process and time constants were calculated.

For the *in vivo* imaging, movement correction was performed using TurboReg plug-in in ImageJ (Thévenaz et al., 1998). The fluorescent signals of all pixels in each ROI was averaged. Two-photon imaging data were smoothened with Kalman stack filter using ImageJ (Kandepu et al., 2008). Data on one-photon wide-field imaging were not filtered. Photobleaching was corrected with spline function in MATLAB, and ΔF/F was calculated with custom-made MATLAB routine. SNR was quantified as peak ΔF/F over the standard deviation of the baseline fluctuation during a 1 s period before stimulus onset.

Statistical analyses were performed with Prism 6.0 software (GraphPad) or KyPlot 5.0 software (Kyenslab). p values < 0.05 was considered as significant. Statistical methods used in the analysis are described in figure legends or experimental procedures. In Figures 5, 6, and 7, we considered that visually-evoked potential was detectable with GEVIs if SNR of optical signals measured with GEVIs was significantly larger than that of EGFP. Number of samples are shown in figure legends.

### DATA AND SOFTWARE AVAILABILITY

The custom MATLAB codes used in this study and analyzed data are available in Mendeley online (https://doi.org/10.17632/8rxrc428bp.1).

## Supplementary Material

Supplementary Figures

## Figures and Tables

**Table:** KEY RESOURCES TABLE

REAGENT or RESOURCE	SOURCE	IDENTIFIER
Chemicals, Peptides, and Recombinant Proteins		
Papain	Worthington Biomedical	LS003120
Poly-L-lysine	BD Biosciences	P4707-50ML
Neurobasal medium	Life Technologies	21103-049
Glutamine	Sigma-Aldrich	G7513-100ML
B27 supplement	Life Technologies	17504044
Minimal essential medium	Life Technologies	11090073
Deposited Data		
Custom-made MATLAB codes and analyzed data	This paper, Mendeley	https://doi.org/10.17632/8rxrc428bp.1
Experimental Models: Organisms/Strains		
Mouse (wild-type, C57BL/6J)	Jackson Laboratory	N/A
Mouse (wild-type, CD-1; ICR)	Charles River	N/A
Recombinant DNA		
pCAG-EGFP	This paper.	N/A
pCAG-ArcLight Q239	Jin et al., 2012	N/A
pCMV-ArcLight Q239	Jin et al., 2012	N/A
pCAG-ArcLight-MT	Kwon et al., 2017	N/A
pCMV-ArcLight-MT	Kwon et al., 2017	N/A
pCAG-ASAP1	St-Pierre et al., 2014	N/A
pCMV-ASAP1	St-Pierre et al., 2014	N/A
pCAG-ASAP2f	Yang et al., 2016	N/A
pCMV-ASAP2f	Yang et al., 2016	N/A
pCAG-ASAP2s	Chamberland et al., 2017	N/A
pCMV-ASAP2s	Chamberland et al., 2017	N/A
pCAG-Ace2N-4AA-mNeon	Gong et al., 2015	N/A
pCMV-Ace2N-4AA-mNeon	Gong et al., 2015	N/A
pCMV-QuasAr2	Hochbaum et al., 2014	N/A
pCMV-Archer1	Flytzanis et al., 2014	N/A
Software and Algorithms		
Electrophysiological recording: PackIO	Watson et al., 2016	https://github.com/apacker83/PackIO
One-photon imaging: HCImage	Hamamatsu Photonics	N/A
Two-photon imaging and electrophysiological recording: Prairie View	Bruker	N/A
Data analysis: ImageJ	NIH	https://imagej.nih.gov/ij/
Data analysis: TurboReg plugin for ImageJ	Thévenaz et al., 1998	http://bigwww.epfl.ch/thevenaz/turboreg/
Data analysis: MATLAB	Math Works	https://www.mathworks.com/
Data analysis: Prism 6.0	GraphPad	N/A
Data analysis: KyPlot 5.0	Kyenslab	http://www.kyenslab.com/en/index.html

## Highlights

- Side-by-side comparison of genetically encoded voltage indicators (GEVIs)
- Side-by-side evaluation of 8 GEVIs in cultured neurons with photon imaging
- Side-by-side evaluation of 4 GEVIs *in vivo* with 1-photon and photon imaging
- Detection of optical field potential (OFP) with GEVIs *in vivo*
