# Supplementary material for: Comparative Evaluation of Genetically Encoded Voltage Indicators
Source: Cell Rep. Author manuscript; Available in PMC 2020 Mar 16. (PMC7075032; doi:10.1016/j.celrep.2018.12.088)
Supplement: Supplementary Figures [file NIHMS1562092-supplement-Supplementary_Figures.pdf]

Cell Reports, Volume *26*

Supplemental Information

Comparative Evaluation

of Genetically Encoded Voltage Indicators

Yuki Bando, Masayuki Sakamoto, Samuel Kim, Inbal Ayzenshtat, and Rafael Yuste

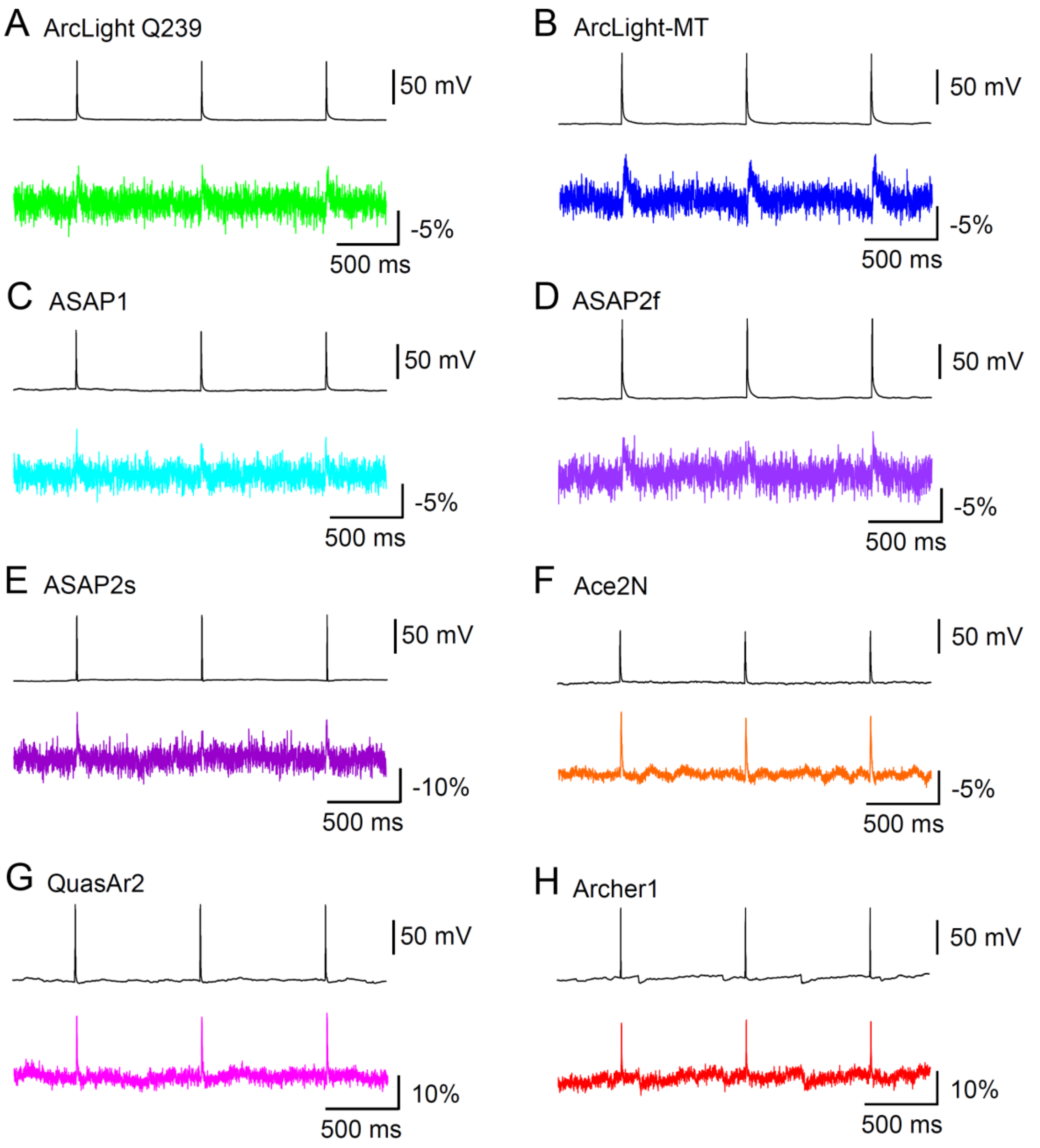

Figure S1. Single-trial recording of APs with GEVIs. Related to Figure 2.

(A-H) Representative traces of ArcLight Q239 (A), ArcLight-MT (B), ASAP1 (C), ASAP2f (D), ASAP2s (E), Ace2N-4AA-mNeon (F), QuasAr2 (G) and Archer1 (H). Upper panels are electrical traces and Lower panels are optical traces. Action potentials were induced with 5 ms current injections.

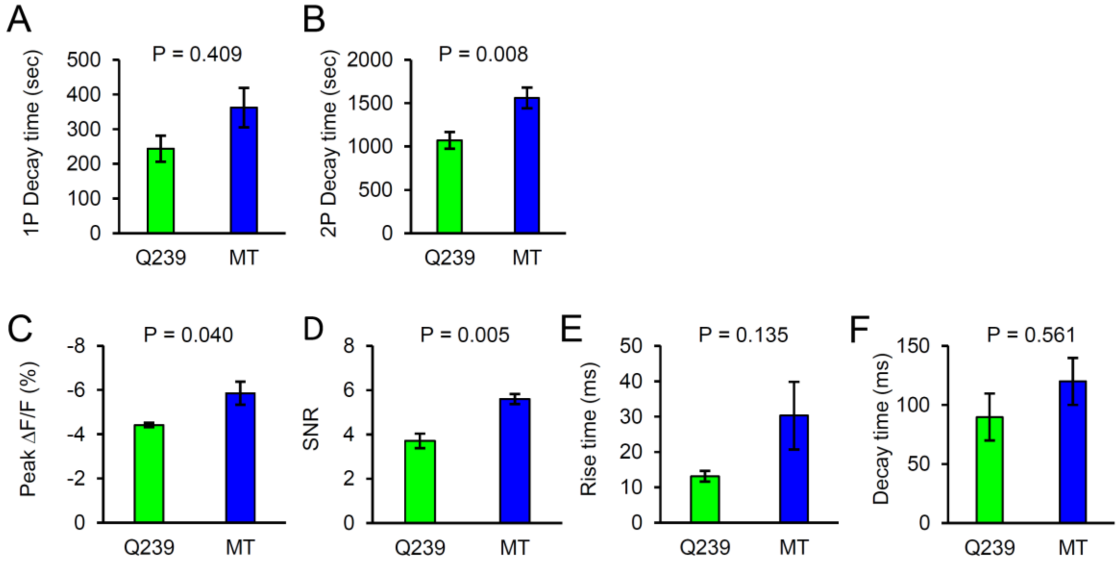

Figure S2. Comparison of ArcLight Q239 and ArcLight-MT. Related to Figures 2 and 4.

(A, B) Comparison of one-photon (A) and two-photon photostability (B). Data are referred from Figure 4. **(C-F)** Performance of ArcLight variants in response to single action potentials. Data are referred from Figure 2. Mean ± S.E.M. are presented. For statistical analysis, two-tailed Student's t-test was performed.

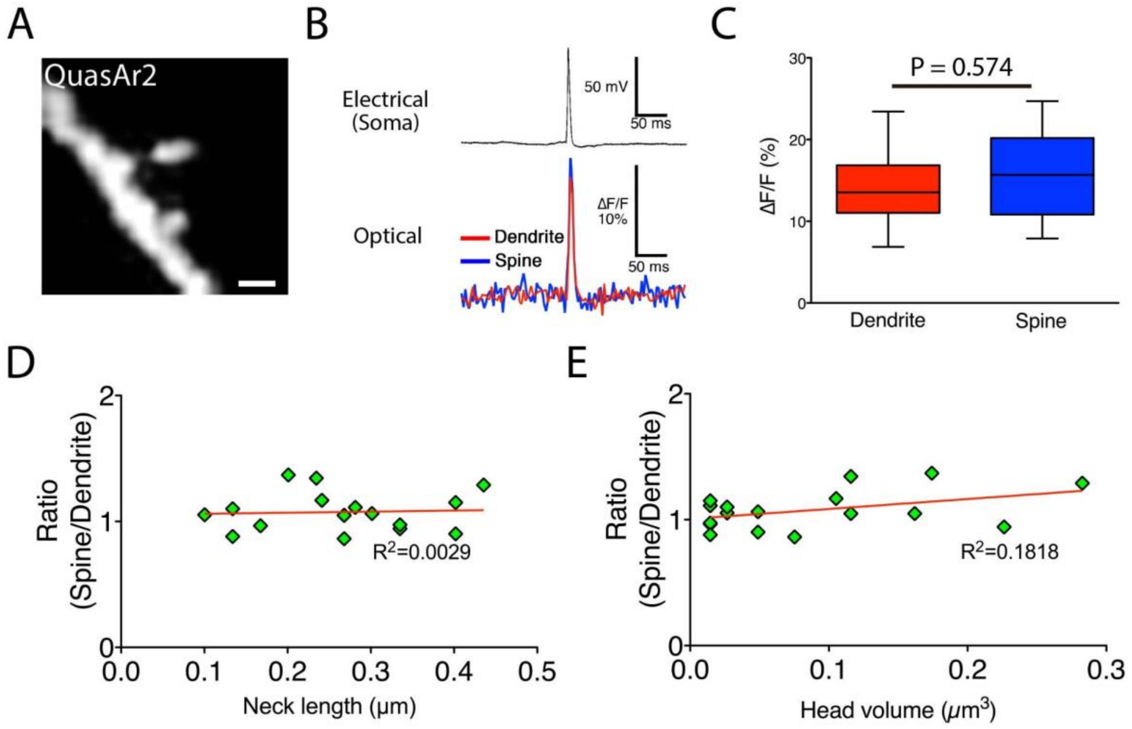

Figure S3. Voltage imaging of dendritic spines with QuasAr2. Related to Figure 2.

(A) A fluorescent image of dendritic spines of a cultured mouse hippocampal neuron

expressing QuasAr2. Scale bar, 1 µm. **(B)** Electrophysiological recording of action potentials induced by current injection in the soma (top) and the average optical waveform (bottom) of QuasAr2 responses in the spine and its parent dendrite (30 trials). Images were taken with and EM-CCD camera (frame rate, 250 Hz). **(C)** Average peak responses to single action potentials for spine and dendrite (n = 17). **(D, E)** Plot of ΔF/F ratio of spine to parent dendrite as a function of spine neck length (D) and spine head volume (E) in response to back-propagating action potentials. N.S. not significant (two-tailed Student's t-test).

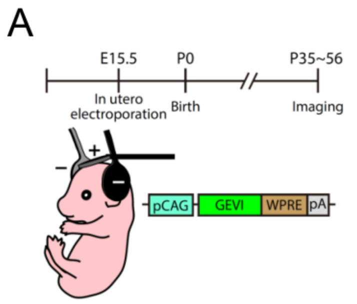

B

	Voltage (V)	Duration (ms)	Interval (ms)	# of pulses	Decay (%)
Poring pulse	50	10	50	3	10
Transferring pulse	15	50	50	3	20

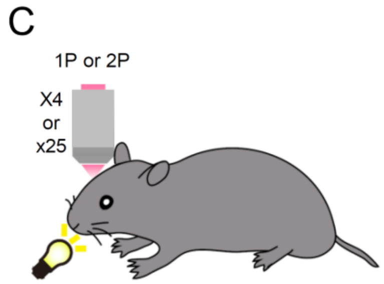

Figure S4. Experimental design of *in vivo* imaging. Related to Figures 5-7.

(A) A scheme of *in utero* electroporation with a triple electrode to target primary visual cortex. **(B)** Parameters for *in utero* electroporation with a triple electrode. **(C)** An experimental design of one- or two-photon voltage imaging *in vivo* of head-fixed and anaesthetized mice.

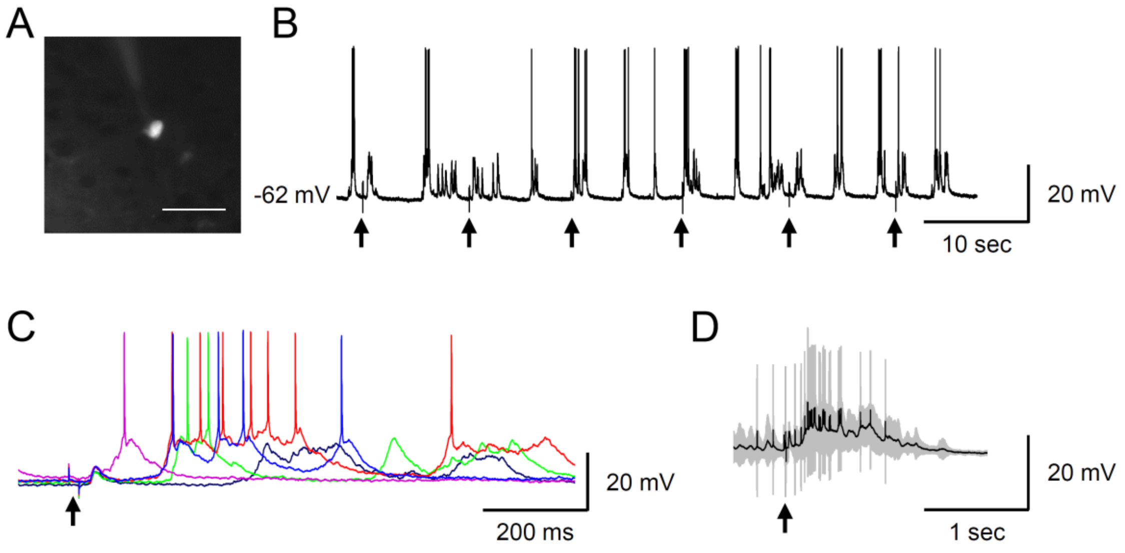

Figure S5. Whole-cell recording of visually-evoked activity from layer 2/3 neurons in the primary visual cortex *in vivo*. Related to Figure 6.

(A) A two-photon image of a recorded pyramidal neuron in layer 2/3 of primary visual cortex. Alexa 594 was filled through a patch pipette. Scale bar, 40 μm. **(B)** Membrane potential dynamics of a neuron shown in (A). Arrows indicate timing of visual stimuli. **(C)** Five examples of visually-evoked electrical activity. Timing of spikes differs among trials. An arrow indicates timing of visual stimulation. **(D)** Mean (a black trace) and S.D. (gray area) of visually-evoked potentials over 10 trials. Action potentials were attenuated, and subthreshold potential was extracted after averaging.

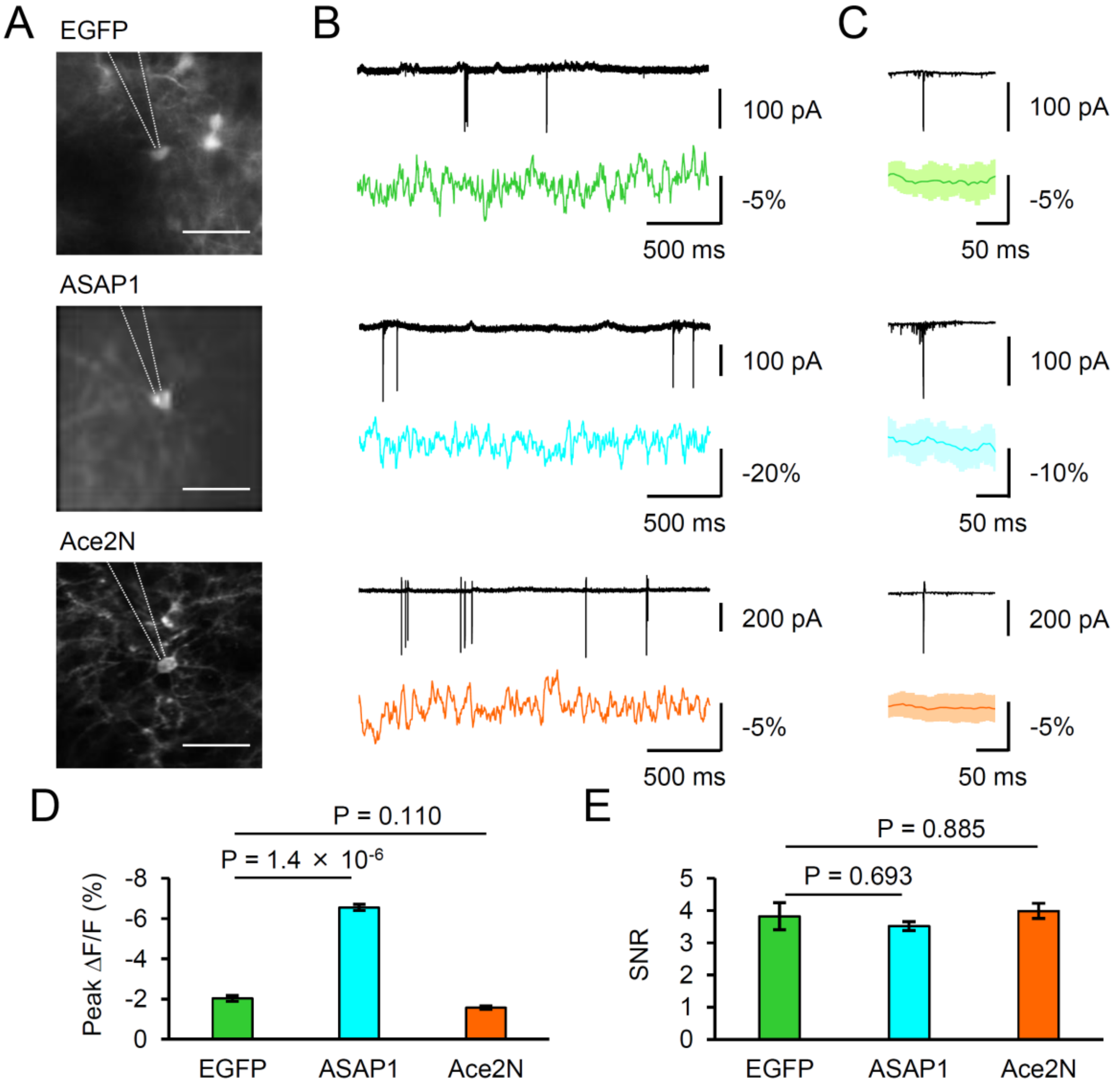

Figure S6. Simultaneous two-photon voltage imaging and cell-attached recording *in vivo*. Related to Figure 6.

(A) Two-photon images of recorded neurons. Dotted lines show position of patch-pipette. Scale bar, 40 µm. **(B)** Electrical (black traces) and optical (colored traces) of the cells shown in (A). **(C)** Spike-triggered average. Mean (black and colored traces) and S.D. (shaded area) are presented. **(D-E)** Peak ΔF/F (D) and SNR (E) of optical response to action potentials. ASAP1 showed larger peak ΔF/F than EGFP, but its SNR was close to EGFP. Ace2N showed similar peak ΔF/F and SNR to EGFP. These results suggest that ASAP1 and Ace2N cannot detect action potentials with two-photon imaging *in vivo*. n = 63 spikes (from 2 cells, 2 mice), 218 spikes (from 3 cells, 2 mice), 296 spikes (from 3 cells, 2 mice). Dunnett test was performed for statistical analysis.

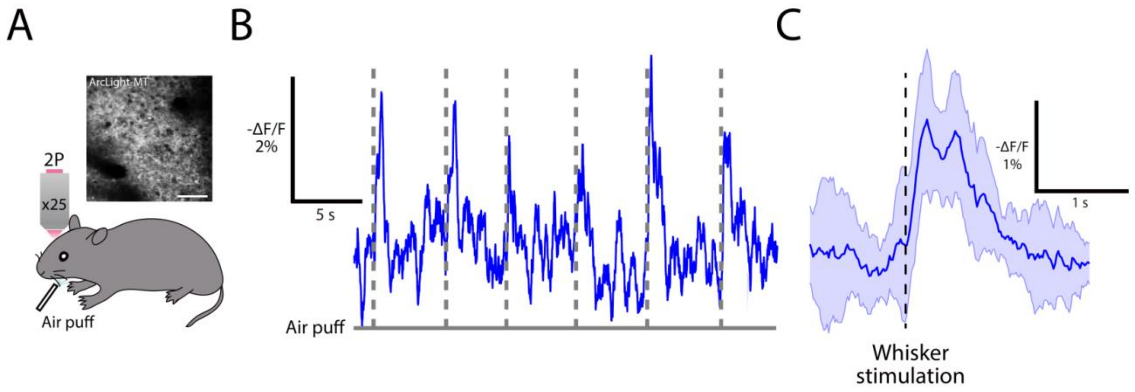

Figure S7. Sensory-evoked optical-field analysis in the barrel cortex at slower scanning speed. Related to Figure 7.

(A) Experimental design. The barrel cortex of head-fixed and anaesthetized mice was imaged. Whiskers were stimulated with air puff for 50 ms, 10 times. A fluorescent image of layer 2/3 neurons in the barrel cortex expressing ArcLight-MT. Scale bar, 50 μm. **(B)** Whisker stimuli-evoked OFP in the field of view shown in (A). Images of 512 x 512 pixels were obtained at 30 Hz. Dashed lines show timing of whisker stimuli. **(C)** Averaged sensory-evoked OFP over 10 trials. Shaded area shows S.D. of the mean.
